# Arts engagement for mental health: A strengths-based opportunity

**DOI:** 10.1371/journal.pmen.0000369

**Published:** 2025-07-07

**Authors:** Alexandra K. Rodriguez

**Affiliations:** 1 University of Florida College of Public Health and Health Professions, Gainesville, Florida, United States of America; 2 University of Florida Center for Arts in Medicine, Gainesville, Florida, United States of America; PLOS: Public Library of Science, UNITED KINGDOM OF GREAT BRITAIN AND NORTHERN IRELAND

## The role of the arts in supporting my own mental health

In October 2020, I faced a near suicide attempt that led to my first hospitalization. My lifelong struggle with severe anxiety and depression had reached an unprecedented peak, manifesting in both psychological and physical ways. My subsequent reality felt insurmountable. At the time, I was in my first year of medical school. While I was not faltering in my coursework, the lack of joy and balance in my life propelled my struggle with mental illness into an unprecedented space. As someone of Puerto Rican heritage, and knowing I was on track to become the first in my family to earn a doctorate, taking a leave of absence from medical school felt like a loss not only for myself but also for my family and broader community.

After recovering and returning home, the challenges remained. I experienced an absence of hope in the weeks and months following. I deeply felt that there was no path forward for me. In hindsight, I can see I was grieving the loss of a path I had envisioned for myself since early childhood. In addition to a perceived loss of purpose, the coming months consisted of new struggles heightened by navigating medication regimens and therapists.

Time spent engaging in the arts were the few distinct moments I can remember during that period that brought me any sense of levity. When I dealt with suicidality, I could not conceptualize a future, let alone one with meaning. Sharing time creating with friends and singing lyrics that resonated with me helped me reframe my lowest period as an inflection point, one from which I could envision both fulfillment and joy.

The opportunity to return to medical school within a two-year window gave me the room to think — and breathe. I chose to use this time to pursue a master’s in public health, a degree I had intended to pursue later in my career. During that first semester, I began to engage in research with the University of Florida’s Center for Arts in Medicine, a place where I found a deep sense of purpose. During my undergraduate studies, I minored in Theatre, but I had never considered how I could merge that passion with my health focused career trajectory. I quickly realized that pursuing research at the intersection of arts and health allowed me to step into spaces as my whole self. In 2021, I engaged in work for the Centers for Disease Control and Prevention under Dr. Jill Sonke where we considered how the arts could be engaged to build both trust and confidence in COVID-19 vaccination [1]. As I continued to collaborate in the Center for Arts in Medicine’s Interdisciplinary Lab, I also had the opportunity to lead a grant funded, mural-based project which sought to shift social perceptions of COVID-19 vaccination, connect students to reliable resources, and directly increase vaccinations rates through a co-location collaboration with UF HealthStreet [2]. As my engagement continued, it became clear how my personal experience with the arts in supporting my mental health helped me to realize an alignment in passion and purpose as an artist and researcher. As I became more engrained in the work, a PhD in public health with a focus on arts and health became a path with intentionality and meaning that I was unable to vision for myself before.

## Spotlighting arts in mental health research and interventions

The professionalization of the arts and health field, which spans from clinical to public health to everyday practices, has made strides this past decade. Namely, the World Health Organization’s review of the field and the epidemiological evidence built out by the EpiArts Lab has catalyzed research within the arts in public health space [3,4]. Relating to mental health specifically, research has shown that adults over the age of 50 who visited cultural venues every few months had a 32% lower risk of developing depression over 10 years [5,6]. Internationally, public health grounded practices, such as arts prescribing, a form of social prescribing, have further mobilized opportunities for mental health support as it connects patients via referrals to arts engagement from their medical providers [7].

Arts for adolescent mental health is a burgeoning sub-field which offers promise both in relation to mental health promotion as well as treatment and management of mental illness [3]. The literature has shown that arts engagement can reduce the risk of developing multiple forms of mental illness, such as depression, in adolescence as well as in older age [5,6]. As someone whose struggle with severe depression and anxiety began in adolescence, the concept that arts engagement can provide support for self-expression and emotion regulation – preventative or rehabilitative strategies for managing forms of mental illness – felt deeply compelling. In fact, recent work has even shown that frequent participation in performance-based art forms predicts higher positive mental health in young adults [8].

For researchers and advocates in the adolescent and young adult mental health space, engaging in arts-based approaches offers the opportunity to augment current practices to support mental health through an asset-based lens. Projects like One Nation/One Project’s Arts for EveryBody campaign have effectively leveraged the evidence in the field to mobilize community centered strategies for arts and health at a national scale [9]. More specifically, through their work, several federally qualified health centers undertook forms of social prescribing where patients with mental health conditions were connected with arts programming as a form of social and emotional support. Additionally, the Jameel Arts & Health WHO collaborating lab has extended this work into community action through their internationally focused policy briefs [10].

Today, I lean into my multiplicity. I am fortunate to be a PhD candidate in public health and a Health Policy Research Scholar Fellow, a program run by Johns Hopkins Bloomberg School of Public Health. My work in advancing arts engagement as a health behavior has led to opportunities such as co-authoring the World Health Organization report centered on how we realize the potential of the arts to support health and wellbeing in the United States [11,12]. Further, and most importantly, my mental health and wellbeing have been supported by the intentionality of my practice, its alignment with my strengths, and by prioritizing the creation of joy for myself and others. Through my engagement with this field, I have found hope in the ways that the arts can support not only direct mental health benefits, but even more broadly strengthen mental health communication, drive access through co-location, foster forms of social connection, and create engaging and inclusive environments.

As we work to address the mental health crisis in the United States and abroad, I hope that we — as mental health professionals, researchers, clinicians, and communities — can collectively consider our challenges while also embracing the arts as a strengths-based approach that offers effective strategies to enhance our current mental health infrastructures.
